# Supplementary material for: Skull-femoral traction followed by osteotomy correction is a safe and effective treatment for severe scoliosis with split cord malformation
Source: PLoS One. 2024 Sep 12;19(9):e0309646. doi: 10.1371/journal.pone.0309646 (PMC11392416; doi:10.1371/journal.pone.0309646)
Supplement: Attachment — Submitted filename: (guo)response_letter_to_the_editor.docx [file pone.0309646.s002.docx]

3-Aug-2024

Manuscript ID: PONE-D-24-23086

Dear Prof. Barry Kweh，

We hereby re-submit our revised manuscript entitled **“Skull-femoral traction followed by osteotomy correction is a safe and effective treatment for severe scoliosis with split cord malformation” (Manuscript ID: PONE-D-24-23086).**

The author's team is honored to hear from the editor and receive thoughtful comments and feedback from the reviewers. We appreciate your valuable advice and have made substantial revisions to our manuscript accordingly.

1.We have carefully compared our manuscript with the template format of your journal and have modified the format of the manuscript to conform as closely as possible to the requirements of your publication.

2.We have supplemented all the raw data required for the research findings in the revised submission files, which have been uploaded as supporting information.

3.All of our authors are in agreement to share our raw data.

4.The corresponding author has completed the ORCID authentication.

5.An ethical statement has been added in the Methods section, explicitly stating that the patient has given consent for the publication of case details.

We have provided point-to-point responses to all the reviewer's questions in the uploaded file “response letter to the reviewers” and made corresponding modifications to the manuscript based on the questions raised by the reviewers. All the revisions we have made with the manuscript are marked in red in the uploaded file “revised manuscript (marked copy)”. The updated version of supporting information is in the file named “revised supporting information”.

We hope our work and effort could move this article closer for publication in your esteemed journal. If you have any additional question or concern, please do not hesitate to contact me at your convenience.

Sincerely,

Tao Guo
