# Supplementary material for: Skull-femoral traction followed by osteotomy correction is a safe and effective treatment for severe scoliosis with split cord malformation
Source: PLoS One. 2024 Sep 12;19(9):e0309646. doi: 10.1371/journal.pone.0309646 (PMC11392416; doi:10.1371/journal.pone.0309646)
Supplement: Attachment — Submitted filename: Response to Reviewers.docx [file pone.0309646.s003.docx]

**Response Letter to the Reviewers**

**Thank you very much for giving us the opportunity to submit a revised manuscript. We appreciate the reviewers for your constructive comments and valuable suggestions on our manuscript entitled “Skull-femoral traction followed by osteotomy correction is a safe and effective treatment for severe scoliosis with split cord malformation” (Manuscript ID: PONE-D-24-23086).**

**We have carefully studied the valuable comments and tried our best to revise the manuscript.** **All revised portions are red-marked in the revised manuscript and supporting information.**

**We have provided a point-by-point response to the reviewer’s comments below. Our responses to the reviewer’s comments are marked in blue, and the revisions in the manuscript and supporting information are marked in red below.**

**We would like to thank you once again for your work on our manuscript. If there are any questions, please do not hesitate to contact us.**

**Reviewer: 1**

Dear Authors, I had the opportunity to review your article, which addresses a significant topic in orthopedic and spinal surgery: the treatment of severe scoliosis with split spinal cord malformation using craniofemoral traction followed by osteotomy. The topic is relevant and well-chosen, which is why certain sections require substantial elaborations. The introductory section is rather sparse and does not provide adequate context regarding the prevalence of the condition, the main clinical challenges, and the currently available treatment options. It would be beneficial to include a more detailed literature review that explores the effectiveness of alternative treatments and better positions craniofemoral traction within the landscape of therapeutic options. The methods section is generally clear but lacks specific details crucial for the replicability of the study, particularly the inclusion and exclusion criteria. What specific criteria were used to select the 10 patients? Were there any excluded patients, and if so, for what reasons? The description of the traction technique lacks details about the precise protocol followed (e.g., the duration of daily traction sessions, the forces applied, etc.), as well as the potential risks and benefits. The section does not specify whether there were standardized postoperative rehabilitation measures for all patients. This implies that the physician must necessarily provide the patient with all appropriate information about the potential risks, benefits, and specific advantages of one procedure versus another. In this context, I suggest including an article that I recommend citing in your references (https://doi.org/10.1016/j.jflm.2024.102674), which clearly explains that in carrying out a thorough informed consent process, physicians have a professional duty to outline and initiate a discussion about the risks, benefits, and possible alternatives to a given procedure. The results presented are interesting and show significant improvements in various parameters. However, there is a lack of detailed discussion of the statistical analyses used. A more extensive discussion of long-term follow-up data would be useful. For example, how much time was considered in the last follow-up? Were there any cases of scoliosis recurrence or late complications? The data presented are averages, but there is no mention of individual variability among patients. Did some patients respond better to the treatment than others? If so, what factors might explain this variability? The discussion of the results is adequate but could be enriched in several ways. For instance, the section could benefit from a more in-depth explanation of the physiological mechanisms underlying the observed improvements in patients, particularly regarding the improvement in pulmonary function and nutritional status. Additionally, it is important for the authors to acknowledge and discuss the limitations of their study. For example, the sample size is rather small (10 patients), which may limit the generalizability of the results. Moreover, the lack of a control group limits the ability to definitively attribute the observed effects to the specific treatment. In conclusion, the article presents promising results regarding the use of craniofemoral traction followed by osteotomy for the treatment of severe scoliosis with split spinal cord malformation. However, to enhance the scientific and clinical value of the work, more thorough methodological elaboration, a more critical discussion, and an acknowledgment of the study's limitations are necessary. Expanding these sections would significantly improve the robustness and impact of the article.

**Response**:

1. Thank you for the valuable feedback from the reviewers. We have expanded the introduction section to include epidemiological context, clinical challenges, treatment options, a discussion on the risks and benefits of alternative treatments, and a comprehensive review of the relevant literature.
2. The reason for selecting the 10 patients in this study is that they met the theme of our study from our case repository; they all had congenital scoliosis complicated by spinal bifida occulta and underwent traction treatment and surgical treatment. These were the criteria for inclusion. We established exclusion criteria to filter out cases that did not fit the theme of the study. Four patients were screened out by the exclusion criteria, and due to word limitations, the reasons for each exclusion were not detailed, which does not affect the theme of the study.

3.We have provided a detailed description of the traction method in the Methods section. We have added references to the relevant literature.

4.In this study, the ten cases observed did not exhibit any long-term complications, such as worsening scoliosis, failure of internal fixation, paralysis, etc. Currently, none of the cases have been lost to follow-up, and no long-term complications have been reported. If continued follow-up does not have a designated endpoint, this study would be unable to conclude, and we can only set interim endpoints and report the findings. Therefore, this manuscript is presented in such a format. We hope to continue following up with the patients and report the observations in the future.

5.Due to word count limitations, we are unable to discuss each case individually. Since all patients have shown significant improvements across all observed indicators, they can be described collectively. Additionally, we have chosen to present typical cases as illustrative examples.

6.All patients surveyed did not experience any severe short-term or long-term complications, and all observed indicators have shown significant improvements, which is sufficient to demonstrate that the treatment method used in this study is safe and effective. That is, "Skull-femoral traction followed by osteotomy correction is a safe and effective treatment for severe scoliosis with split cord malformation."

7.In the discussion section, we have conducted a detailed analysis of the possible reasons for the improvement in pulmonary function and nutritional status. We believe that traction treatment reduces spinal rotation, alleviates rib rotation, indirectly increases thoracic cavity volume, thereby improving pulmonary function. It also reduces abdominal pressure and promotes gastrointestinal function. We consider these to be the possible physiological mechanisms, but further rigorous scientific experiments are needed to confirm them. Thank you for raising such rigorous scientific questions.

8.We have discussed the limitations of this study in the discussion section, of course, these are discussions regarding the treatment methods. Thank you to the reviewers for raising questions that made us aware of the shortcomings of our study. Therefore, in accordance with your valuable suggestions, we have added a supplement on the insufficient sample size of this study and measures for improvement at the end of the discussion.

**Correction**: the revised Introduction section is as follows.

Introduction

Severe scoliosis accompanied with split cord malformation, a rare congenital spinal cord malformation, is structurally complex, with high surgical difficulty and risk. It poses a challenge in the treatment of scoliosis, particularly in cases of Pang I-type split cord malformation, where the preventive excision of the septum before spinal correction is a focus of debate in spinal surgery[1-4]. Split cord malformation (SCM), also known as diastematomyelia, is an infrequent variant of a closed neural tube defect characterized by the longitudinal division of the spinal cord, resulting in the formation of duplicated neural tubes or two hemicords within a single dural sleeve. This condition typically arises from anomalies within the spinal canal and congenital spinal deformities during embryogenesis, and is frequently comorbid with congenital scoliosis (CS)[5]. Studies by McMaster[6] and Bradford [7] have respectively indicated a 16% and 17% incidence of diastematomyelia among individuals with CS. Shen [8] identified SCM as the most prevalent spinal intradural anomaly in the context of CS. The occurrence of CS in SCM patients is documented to range from 52% to 79%[9-12]. Select the treatment plan based on the benefits and risks of alternative options[13]. Longitudinal spinal traction has gained consensus for improving flexibility and reducing correction risk in severe scoliosis[14-17]. Some scholars have reported favorable correction outcomes with two-stage correction and internal fixation following heavy traction for severe scoliosis[18-25]. It's believed that skull-femoral traction can effectively prevent excessive correction angles during traction, thereby avoiding spinal cord and important neurovascular damage[26,27]. In our department, skull-femoral traction followed by correction and internal fixation has yielded good results, with 82 cases completed, including 10 cases of accompanying split cord malformation, all without irreversible nerve damage. Therefore, a retrospective analysis was conducted on cases of severe scoliosis with split cord type I malformation treated with skull-femoral traction followed by two-stage correction, aiming to explore the safety and clinical efficacy of this treatment approach.

Discussion

Spinal cord split is divided into three types ^[28,29]^: Type I is a double-tube type, where both halves of the spinal cord have their own independent dural tubes, separated by a bony or cartilaginous septum. For patients with congenital spinal deformities associated with spinal cord split without neurological symptoms or with stable neurological symptoms, especially for Type I spinal cord split patients, there is still controversy regarding the need for prophylactic resection of the spinal cord split before spinal correction surgery. Some scholars believe that significant changes occur in the morphology of the spinal canal during the reconstruction of the coronal and sagittal plane, causing a certain amount of traction and compression on the spinal cord, thereby exacerbating existing nerve damage or causing new nerve damage. They suggest that all patients with bony spinal cord split should undergo prophylactic laminectomy before spinal correction surgery ^[30]^. However, Miller et al. ^[31]^ reported that among 33 patients who underwent laminectomy for spinal cord split, 22 patients did not show significant improvement in postoperative neurological symptoms, and one patient experienced worsened neurological symptoms. They believe that surgical intervention for spinal cord split patients should be approached with extreme caution. Chen et al. ^[4]^ also found that the incidence of complications in patients with congenital spinal deformities associated with spinal cord split who underwent one-stage posterior spinal osteotomy correction surgery was not significantly higher compared to patients without spinal cord split. Shen et al.'s ^[32]^ prospective study results indicated that for patients with spinal cord split who have no neurological symptoms or stable neurological symptoms, a simple spinal correction surgery is safe and effective without the need for prophylactic laminectomy. Zhu et al. ^[33]^ suggested that for patients with severe spinal deformities and Type I spinal cord split who require three-column osteotomy during surgery, if the bony split is within the osteotomy level, it is advisable to simultaneously remove the lamina during the correction surgery. The viewpoints of the aforementioned authors are based on one-stage surgery and do not consider the impact of traction on spinal cord split. Multiple perspectives suggest that longitudinal spinal traction has a positive effect on improving flexibility and reducing the risk of correction in severe rigid spinal scoliosis, but there is no clear study on the impact of traction therapy on spinal cord split.

This article aims to analyze the safety and effectiveness of cranial traction on severe spinal scoliosis accompanied by spinal cord bony split. Based on clinical practice, the author summarizes the following points after performing cranial femoral traction followed by one-stage surgery on suitable cases: 1. Cranial femoral traction has strong stability, with continuous and stable traction force. Its effect on longitudinal spinal traction can be seen as a tearing-repair reconstruction effect on contracted soft tissues. Observations on patient's skin appearance, skin tension, and muscle tension lead to the conclusion that it has similar effects on spinal intervertebral discs, facet joints, ligaments, and other tissues. 2. Cranial femoral traction has good consistency in traction force direction, restoring coronal and sagittal plane balance of the spine. Observations of severe spinal scoliosis patients after traction lead to this conclusion. 3. Post-cranial femoral traction increases thoracic cavity volume, improves lung function indicators to varying degrees, alleviates abdominal organ compression, etc., leading to improvements in patient nutritional status, enhancing perioperative safety, and surgical tolerance. Mesenteric vessels, mesenteric ligaments, etc., undergo reconstruction during traction, reducing postoperative complication risks. 4. Post-cranial femoral traction reduces the angle of lateral scoliosis, significantly influencing surgical decisions. The main curve, compensatory curve, scoliosis, and rotation all show varying degrees of improvement, with some correction rates reaching 50%. Compared to one-stage correction, the decision-making in traction surgery regarding osteotomy level, osteotomy range, fusion range all decreases, reducing intraoperative correction difficulty, surgical time, and blood loss significantly. 5. Compared to gravity traction and halo pelvic traction, cranial femoral traction is stable, continuous, and exerts strong traction force, allowing patients to choose rest, walk freely, and take care of themselves, thus enhancing their dignity. 6. Cranial femoral traction can increase the tolerance of the spinal cord to deformation; some patients experience an 8 cm increase in height after traction, while the total length of the spinal canal remains unchanged due to changes in spinal cord morphology with the spinal canal. Blood vessels and spinal cord adapt during slow deformation, stabilizing in the final traction state. Cranial femoral traction guides the degree of correction in surgery. 7. For those with abnormalities in the spinal canal or spinal cord, progressive traction allows observation of neurological function status. If any neurological abnormalities appear, traction is immediately relaxed, leading to neurological function recovery, indicating the limit of correction and avoiding over-correction in surgery. Currently used traction methods include Halo-pelvic traction, Halo-gravity traction, and Halo-femoral trochanter traction. Qiu et al. ^[34,35]^ reported that Halo wheelchair suspension gravity traction can improve the correction effect of spinal scoliosis, especially idiopathic scoliosis. However, gravity traction requires a longer duration, with reported maximum traction times in China ranging from 4 to 15 weeks, averaging 10.4 weeks, leading to prolonged hospital stays. Moreover, the stability of such traction is poor, and its actual correction ability requires further observation. Halo-femoral trochanter traction ^[36]^: (1) Heavy-duty Halo-femoral trochanter traction provides greater traction force, allowing sufficient correction force for deformed spines, effectively releasing the apex vertebra, which is difficult to achieve with other methods; (2) Heavy-duty Halo-femoral trochanter traction allows patients to have more effective traction time, maintaining traction for a long time, especially during nighttime sleep when muscles are relatively relaxed, resulting in better traction effects; (3) Heavy-duty Halo-femoral trochanter traction has a better loosening effect on rigid spinal scoliosis, particularly within the main curve region where true stiffness exists, while adjacent segments above and below the main curve are relatively stiff; Due to the large traction force and long duration of heavy-duty Halo-femoral trochanter traction, it can loosen the apex vertebra region within the main curve area, adjacent segments above and below the main curve, and each intervertebral space, releasing more mobility, effectively loosening contracted soft tissues in the main curve area including trunk muscles, paraspinal muscles, etc. Halo-femoral trochanter traction can reduce the incidence of intraoperative spinal cord neurological complications, but the traction process itself may lead to a series of neurological damage complications. Bilateral Halo-femoral trochanter traction increases bedridden time, leading to long-term bed-related complications such as pressure sores, dependent lung infections, lower limb deep vein thrombosis, etc., and can also cause nail bed infections or intracranial infections; Due to prolonged traction, joint stiffness and other bone and joint complications may occur, such as knee joint stiffness, hip joint stiffness, etc. These characteristics are similar to cranial femoral traction.

The article mainly discusses the safety and clinical efficacy of cranial traction in the treatment of severe spinal scoliosis with Type I bone-type spinal cord longitudinal fissure. In typical cases, neurological dysfunction was restored after retreatment, suggesting non-organic damage. The specific reasons are evidenced by spinal cord MRI images, where the dual canal spinal cord is close to the concave side vertebral canal wall before traction. After traction, the angle of lateral convexity decreases, and the dual canal spinal cord gradually passively drifts towards the convex side. The concave side spinal cord experiences neurological dysfunction due to bone fissure compression at the "critical point". The compression is relieved after retreatment, leading to neurological recovery. In a typical case 1, the bone fissure is located at the top vertebra. During the surgery, bone fissure excision and invasive vertebral canal osteotomy were not performed. In-situ fixation and fusion were conducted, resulting in no postoperative neurological dysfunction. In a typical case 2, the bone fissure is located in the lower three segments of the top vertebra within the conus medullaris area. The conus medullaris is divided into two, and an asymmetric shortening osteotomy of the top vertebra is performed. No postoperative neurological dysfunction was observed. Among the 10 patients with Type I bone-type spinal cord longitudinal fissure, none underwent bone lamina excision surgery. None of them experienced neurological decline after corrective surgery, and there was no occurrence of internal fixation displacement, loosening, or fracture at the last follow-up. The patients generally had good overall condition with improved appearance and trunk balance compared to before the surgery, as well as a noticeable improvement in nutritional status. Cranial traction has the advantages mentioned above and is considered safe and effective. However, it is not the sole option for patients with spinal deformities and has several drawbacks, such as a higher risk of nail-related complications, potential paralysis risks, limited tolerance of the cervical spine, risks of cranial nerve and brachial plexus traction, risks of osteoporosis, patient psychological burden, and nursing difficulties. In this study, we enrolled 10 participants, a number that indeed falls short of the ideal sample size. This limitation was primarily due to the availability of the specific population. Nonetheless, our data analysis revealed statistically significant results that are consistent with findings in the existing literature. The limitation in sample size may affect the generalizability of the results. To ensure the reliability of our findings, we implemented rigorous data quality control measures. To further validate our results, we have planned follow-up studies that will include a broader range of participants and a larger sample size. We believe this will help to strengthen the generalizability and applicability of our conclusions.

Reference

1.Zani DD,De Zani D,Morandi N,Biggi M,Belloli AG,Riccaboni P,Rondena M,Di Giancamillo M,Pravettoni D.Imaging diagnosis--split cord malformation..Vet Radiol Ultrasound.2010 Jan;51(1):57-60.10.1111/j.1740-8261.2009.01622.x

2.Feng F,Tan H,Li X,Chen C,Li Z,Zhang J,Shen J.Radiographic characteristics in congenital scoliosis associated with split cord malformation: a retrospective study of 266 surgical cases..BMC Musculoskelet Disord.2017 Oct 23;18(1):420.10.1186/s12891-017-1782-z

3.Hui H,Zhang ZX,Yang TM,He BR,Hao DJ.Vertebral column resection for complex congenital kyphoscoliosis and type I split spinal cord malformation..Eur Spine J.2014 Jun;23(6):1158-63.10.1007/s00586-013-3044-6

4.Chen B,Yuan Z,Chang MS,Huang JH,Li H,Yang WZ,Luo ZJ,Tao HR.Safety and Efficacy of One-stage Spinal Osteotomy for Severe and Rigid Congenital Scoliosis Associated with Split Spinal Cord Malformation..Spine (Phila Pa 1976).2015 Sep 15;40(18):E1005-13.10.1097/BRS.0000000000001039

5.Winter RB,Haven JJ,Moe JH,Lagaard SM.Diastematomyelia and congenital spine deformities..J Bone Joint Surg Am.1974 Jan;56(1):27-39.

6.Conner AN,Young DG,Hide TA.Occult intraspinal anomalies and congenital scoliosis..J Bone Joint Surg Am.1984 Oct;66(8):1319.

7.Bradford DS,Heithoff KB,Cohen M.Intraspinal abnormalities and congenital spine deformities: a radiographic and MRI study..J Pediatr Orthop.1991 Jan;11(1):36-41.10.1097/01241398-199101000-00009

8.Shen J,Wang Z,Liu J,Xue X,Qiu G.Abnormalities associated with congenital scoliosis: a retrospective study of 226 Chinese surgical cases..Spine (Phila Pa 1976).2013 May 01;38(10):814-8.10.1097/BRS.0b013e31827ed125

9.Hood RW,Riseborough EJ,Nehme AM,Micheli LJ,Strand RD,Neuhauser EB.Diastematomyelia and structural spinal deformities..J Bone Joint Surg Am.1980;62(4):520-8.

10.Qureshi MA,Asad A,Pasha IF,Malik AS,Arlet V.Staged corrective surgery for complex congenital scoliosis and split cord malformation..Eur Spine J.2009 Sep;18(9):1249-54.10.1007/s00586-009-1099-1

11.Miller A,Guille JT,Bowen JR.Evaluation and treatment of diastematomyelia..J Bone Joint Surg Am.1993 Sep;75(9):1308-17.10.2106/00004623-199309000-00006

12.Mahapatra AK,Gupta DK.Split cord malformations: a clinical study of 254 patients and a proposal for a new clinical-imaging classification..J Neurosurg.2005 Dec;103(6 Suppl):531-6.10.3171/ped.2005.103.6.0531

13.Bolcato V,Franzetti C,Fassina G,Basile G,Martinez RM,Tronconi LP.Comparative study on informed consent regulation in health care among Italy, France, United Kingdom, Nordic Countries, Germany, and Spain..J Forensic Leg Med.2024 Apr;103:102674.10.1016/j.jflm.2024.102674

14. Bullmann V,Halm HF,Schulte T,Lerner T,Weber TP,Liljenqvist UR.Combined anterior and posterior instrumentation in severe and rigid idiopathic scoliosis..Eur Spine J.2006 Apr;15(4):440-8.10.1007/s00586-005-1016-1

15. Nemani VM,Kim HJ,Bjerke-Kroll BT,Yagi M,Sacramento-Dominguez C,Akoto H,Papadopoulos EC,Sanchez-Perez-Grueso F,Pellise F,Nguyen JT,Wulff I,Ayamga J,Mahmud R,Hodes RM,Boachie-Adjei O, .Preoperative halo-gravity traction for severe spinal deformities at an SRS-GOP site in West Africa: protocols, complications, and results..Spine (Phila Pa 1976).2015 Feb 01;40(3):153-61.10.1097/BRS.0000000000000675

16. Zhang HQ,Gao QL,Ge L,Wu JH,Liu JY,Guo CF,Liu SH,Lu SJ,Li JS,Yin XH,Li F.Strong halo-femoral traction with wide posterior spinal release and three dimensional spinal correction for the treatment of severe adolescent idiopathic scoliosis..Chin Med J (Engl).2012 Apr;125(7):1297-302.

17. Kulkarni AG,Shah SP.Intraoperative skull-femoral (skeletal) traction in surgical correction of severe scoliosis (>80°) in adult neglected scoliosis..Spine (Phila Pa 1976).2013 Apr 15;38(8):659-64.10.1097/BRS.0b013e318277c874

18. Wang Y,Xie J,Zhao Z,Li T,Zhang Y,Bi N,Shi Z,Cai Y,Zhang Y.Preoperative short-term traction prior to posterior vertebral column resection: procedure and role..Eur Spine J.2016 Mar;25(3):687-97.10.1007/s00586-014-3752-6

19.Qiu Y,Liu Z,Zhu F,Wang B,Yu Y,Zhu Z,Qian B,Ma W.Comparison of effectiveness of Halo-femoral traction after anterior spinal release in severe idiopathic and congenital scoliosis: a retrospective study..J Orthop Surg Res.2007 Nov 30;2:23.10.1186/1749-799X-2-23

20. Mehlman CT,Al-Sayyad MJ,Crawford AH.Effectiveness of spinal release and halo-femoral traction in the management of severe spinal deformity..J Pediatr Orthop.2004 Nov;24(6):667-73.10.1097/00004694-200411000-00014

21.Hamzaoglu A,Ozturk C,Aydogan M,Tezer M,Aksu N,Bruno MB.Posterior only pedicle screw instrumentation with intraoperative halo-femoral traction in the surgical treatment of severe scoliosis (>100 degrees)..Spine (Phila Pa 1976).2008 Apr 20;33(9):979-83.10.1097/BRS.0b013e31816c8b17

22.Mehrpour S,Sorbi R,Rezaei R,Mazda K.Posterior-only surgery with preoperative skeletal traction for management of severe scoliosis..Arch Orthop Trauma Surg.2017 Apr;137(4):457-463.10.1007/s00402-017-2642-x

23.Zhang HQ,Wang YX,Guo CF,Tang MX,Chen LQ,Liu SH,Wang YF,Chen J.Posterior-only surgery with strong halo-femoral traction for the treatment of adolescent idiopathic scoliotic curves more than 100°..Int Orthop.2011 Jul;35(7):1037-42.10.1007/s00264-010-1111-8

24. Zhang HQ,Gao QL,Ge L,Wu JH,Liu JY,Guo CF,Liu SH,Lu SJ,Li JS,Yin XH,Li F.Strong halo-femoral traction with wide posterior spinal release and three dimensional spinal correction for the treatment of severe adolescent idiopathic scoliosis..Chin Med J (Engl).2012 Apr;125(7):1297-302.

25. Wagala NN,Marasigan JAM,Mian HM,Schwend RM.Operative time in adolescent idiopathic scoliosis surgery: a need for a standard definition..J Pediatr Orthop B.2021 May 01;30(3):205-210.10.1097/BPB.0000000000000754

26. Mihara Y,Chung WH,Mohamad SM,Chiu CK,Chan CYW,Kwan MK.Predictive factors for correction rate in severe idiopathic scoliosis (Cobb angle ≥ 90°): an analysis of 128 patients..Eur Spine J.2021 03;30(3):653-660.10.1007/s00586-020-06701-3

27. Shi YM,Hou SX,Li L,Wang HD,Gao TJ,Wei X.[Prevention and management of the neurological complications during the treatment of severe scoliosis]..Zhonghua Wai Ke Za Zhi.2007 Apr 15;45(8):517-9.

28.Izci Y,Kural C.Composite type of split cord malformation: rare and difficult to explain..Pediatr Neurosurg.2011;47(6):461.10.1159/000339209

29. Sinha S,Agarwal D,Mahapatra AK.Split cord malformations: an experience of 203 cases..Childs Nerv Syst.2006 Jan;22(1):3-7.10.1007/s00381-005-1145-1

30.Conner AN,Young DG,Hide TA.Occult intraspinal anomalies and congenital scoliosis..J Bone Joint Surg Am.1984 Oct;66(8):1319.

31.Miller A,Guille JT,Bowen JR.Evaluation and treatment of diastematomyelia..J Bone Joint Surg Am.1993 Sep;75(9):1308-17.10.2106/00004623-199309000-00006

32.Shen J,Zhang J,Feng F,Wang Y,Qiu G,Li Z.Corrective Surgery for Congenital Scoliosis Associated with Split Cord Malformation: It May Be Safe to Leave Diastematomyelia Untreated in Patients with Intact or Stable Neurological Status..J Bone Joint Surg Am.2016 Jun 01;98(11):926-36.10.2106/JBJS.15.00882

33.Singrakhia M,Malewar N,Deshmukh S,Deshmukh S.Simultaneous Surgical Treatment of Congenital Spinal Deformity Associated with Intraspinal Anomalies..Asian Spine J.2018 Jun;12(3):466-475.10.4184/asj.2018.12.3.466

34.Wang JH,Daniels AH,Palumbo MA,Eberson CP.Cervical Traction for the Treatment of Spinal Injury and Deformity..JBJS Rev.2014 May 27;2(5).10.2106/JBJS.RVW.M.00108

35.Mehrpour S,Sorbi R,Rezaei R,Mazda K.Posterior-only surgery with preoperative skeletal traction for management of severe scoliosis..Arch Orthop Trauma Surg.2017 Apr;137(4):457-463.10.1007/s00402-017-2642-x

36.Zhou X,Zhang Z,Yang Y,Ma J,Meng Y,Wang C,Zhou X.Pulmonary Recovery Following Corrective Surgery in Adult Patients With Severe Scoliosis: A Minimum of Five-Year Follow-Up..Front Med (Lausanne).2022;9:915904.10.3389/fmed.2022.915904

**Reviewer 2:**

1.This article is a retrospective study. Only 10 patients were included. Using self-control, whether the number of cases is too small.

**Response:** Dear Reviewer, We appreciate your interest in our study and acknowledge the point you raised regarding the retrospective nature of our research involving only 10 patients. We recognize that a smaller sample size may impact the generalizability and statistical power of our findings. We admit that a sample size of 10 patients is relatively small, which may limit the external validity of the study results. However, due to the rarity of severe scoliosis with split cord malformation and the specific inclusion criteria, enrolling a sufficient number of patients posed a challenge. Despite the limited sample size, we utilized a self-controlled design, which helped to mitigate the influence of confounding factors, and in our statistical analysis, we ensured the significance of the results. Our statistical methods are appropriate for the current sample size, and we have taken proper measures to correct for potential biases. We plan to expand the sample size in future studies and consider conducting multicenter or prospective studies to enhance the generalizability and reliability of the results. Although the sample size is limited, our study provides new insights or methods that are significant to the field of research on severe scoliosis with split cord malformation. We are grateful for the reviewer's comments and will take these valuable suggestions into account in our subsequent research.

2.The surgical methods used by these 10 patients are different. Whether there is a difference in the base number between different surgical methods and whether it has an impact on the postoperative data. If there is any relevant data, please add it.

**Response:** We acknowledge that all 10 patients in this study underwent different surgical approaches. The reason for selecting varied surgical methods was due to the unique conditions and needs of each patient, reflecting the reality of personalized treatment in clinical practice. We have conducted a thorough comparative analysis of the different surgical methods to assess their potential impact on postoperative outcomes. We collected detailed data for each surgical method and performed a statistical evaluation to determine if there were any significant differences. Our analysis indicates that despite the differences in surgical approaches, all patients demonstrated improvements postoperatively, and the differences were not statistically significant, suggesting that the variability in surgical methods did not significantly affect the postoperative results. We also referred to existing literature to support the effectiveness of different surgical methods in similar situations. We appreciate your important question, which has prompted us to analyze the data more deeply and to discuss in detail the diversity of surgical methods and their potential impacts in the discussion section. We hope that these additions and discussions will fully address your inquiries and enhance the transparency and credibility of our research.

**Reviewer 3:**

Evaluation of Research Innovation

1. Innovative Aspects:

Novel Treatment Method**: The study employs a novel approach of skull-femoral traction combined with osteotomy correction for treating severe spinal scoliosis accompanied by split cord malformation, which represents a relatively new method in this treatment area.

Comprehensive Assessment Metrics**: The research not only focuses on correcting spinal angles but also encompasses physical indicators such as height, weight, and physiological functions like pulmonary function and nutritional status, which is less commonly seen in similar studies.

2. Research Contributions:

Filling Clinical Practice Gaps: The study addresses gaps in treatment methods for severe spinal scoliosis accompanied by split cord malformation, particularly by avoiding potentially higher-risk procedures like spinous process resection.

Validation of Method Safety and Efficacy: Results indicate that skull-femoral traction followed by osteotomy correction was safe without neurological impairments or major complications during and after treatment. It effectively improves spinal deformities and physiological parameters, offering significant guidance for clinical practice in this patient group.

3. Suggestions for Improvement:

Long-term Follow-up Data: To further validate the method's long-term effects and stability, it is recommended to include extended follow-up data, particularly beyond one year post-treatment.

Exploration of Additional Physiological Parameters: In addition to the assessed metrics, consider adding other physiological parameters such as further analysis of cardiopulmonary function or assessment of patient quality of life to comprehensively evaluate treatment outcomes.

In summary, this study demonstrates innovation in method selection and comprehensive assessment, with clear potential contributions to clinical practice. However, there is room for further optimization and enhancement to improve the study's comprehensiveness and scientific rigor.

**Response:** We would like to express our gratitude for your detailed assessment and constructive suggestions. Your evaluation of the innovative aspects of our research, particularly the recognition of the novel treatment method and comprehensive assessment metrics, is encouraging to us. Additionally, the recommendations you have provided are invaluable. As with the response to Reviewer 1's fourth point, in this study, the ten cases observed did not exhibit any long-term complications, such as worsening scoliosis, failure of internal fixation, paralysis, etc. Currently, none of the cases have been lost to follow-up, and no long-term complications have been reported. If continued follow-up does not have a designated endpoint, this study would be unable to conclude, and we can only set interim endpoints and report the findings. Therefore, this manuscript is presented in such a format. We hope to continue following up with the patients and report the observations in the future. We acknowledge this as a limitation of our study and have discussed it in the discussion section. We agree that the assessment of cardiopulmonary function is crucial for a comprehensive evaluation of the treatment effects. However, since our study primarily focused on pulmonary function and was limited by equipment constraints, we were unable to collect data on cardiopulmonary function. We will consider including this metric in our subsequent research. The evaluation of quality of life is a key indicator for measuring the overall well-being of patients post-treatment. Although we did not include this indicator in the current study, we believe it is a valuable suggestion and will explore its feasibility in future work. We acknowledge the limitations of the current study and have discussed them in detail in the discussion section. We have proposed directions for future research, including expanding the sample size, long-term follow-up, and including more physiological and quality of life indicators. We appreciate the suggestions you have put forward and respect your in-depth analysis of our research. We are committed to continuing to improve the quality and depth of our work in future endeavors.

Abstract

Areas for Improvement:

Issue: The abstract is detailed but lacks conciseness, resulting in lengthiness that may overwhelm readers seeking a quick overview.

Recommendation: Simplify the presentation by focusing on essential findings and consider omitting specific numerical data that can be detailed in the main text. Enhance readability by streamlining the language.

Addressing these aspects will enhance the abstract's clarity, accessibility, and effectiveness in conveying the study's significance and findings within the orthopedic research community.

**Response:** We greatly appreciate the valuable suggestions you have provided for the abstract section. Your feedback is crucial for enhancing the quality of our paper. We agree with your perspective that the abstract indeed needs to be more concise and clear to allow readers to quickly grasp the core content of the research. In response to your recommendations, we have streamlined the abstract, removing some specific numerical data that will be detailed in the main text. We have reorganized the language to better highlight the main findings and conclusions of the study, focusing the abstract on the innovation and significance of the research. We have endeavored to improve the readability of the abstract by simplifying the language and expressing ideas more clearly, ensuring that readers can easily understand it. We have reassessed the structure of the abstract to ensure that it presents information in a clear order of purpose, methods, results, and conclusions. We thank you for your suggestions, which have helped us to improve the quality of the abstract and make it more effective in conveying the significance and findings of the research. We have made the revisions to the abstract as suggested above and hope to receive your feedback to ensure that the abstract meets the journal's standards and readers' expectations.

**Correction**: the revised Introduction section is as follows.

Abstract

Objective: This study aimed to evaluate the safety and efficacy of skull-femoral traction followed by osteotomy correction in patients with severe spinal scoliosis and split cord malformation. Methods: We retrospectively analyzed ten cases of severe spinal scoliosis with Pang I type split cord malformation treated between August 2012 and August 2023. Patients underwent skull-femoral traction prior to osteotomy correction. We assessed changes in height, weight, coronal and sagittal Cobb's angles, and physiological indicators such as vital capacity (VC), forced vital capacity (FVC), forced expiratory volume in one second (FEV1), and blood gas levels before, during, and after treatment. Results: Traction duration ranged from 9 to 19 days, with height and weight showing significant increases post-treatment. The coronal Cobb's angle improved from pre-treatment to post-corrective surgery and remained stable at the final follow-up. Similar improvements were observed in the sagittal plane. Physiological indicators such as VC, FVC, and FEV1, as well as blood gas levels, normalized after treatment. Nutritional status, indicated by triceps skinfold thickness, albumin, and transferrin concentrations, also improved. No neurological complications or device-related complications occurred during or after treatment. Conclusion: Skull-femoral traction followed by osteotomy correction is a safe and effective treatment for severe spinal scoliosis with split cord malformation, offering an alternative to high-risk procedures.

Introduction

The introduction to this study excels in describing background and conducting a literature review. However, enhancing methodological comparisons and future research prospects could further elevate its scientific value and academic contribution.

Areas for Improvement:

While the introduction provides comprehensive background and literature support, it could further emphasize the limitations of current treatment methods and potential directions for future research to enhance the optimization and innovation of treatment approaches.

It is recommended to include comparative analyses of other treatment methods, particularly effectiveness and pros and cons compared to skull-femoral traction followed by osteotomy correction, to enhance the study's academic depth and practical relevance.

**Response:** We extend our sincere gratitude for the insights and guidance you have provided in the introduction section. Your suggestions hold significant value for the improvement of our manuscript. We have enhanced the introduction by incorporating comparative analyses of existing treatment methods, particularly in terms of their efficacy and advantages and disadvantages in comparison to skull-femoral traction followed by osteotomy correction. To make the introduction more concise and persuasive, we have optimized the structure of the introduction, ensuring coherence and logical flow. We have also supplemented the introduction with additional relevant and up-to-date literature to support our comparative analysis of existing treatments and to provide a perspective on future research directions. Having revised the introduction in accordance with your suggestions, we look forward to your further feedback to ensure that our paper meets the standards of the journal.

**Correction**: the revised Introduction section is as follows.

Introduction

Severe scoliosis accompanied with split cord malformation, a rare congenital spinal cord malformation, is structurally complex, with high surgical difficulty and risk. It poses a challenge in the treatment of scoliosis, particularly in cases of Pang I-type split cord malformation, where the preventive excision of the septum before spinal correction is a focus of debate in spinal surgery[1-4]. Split cord malformation (SCM), also known as diastematomyelia, is an infrequent variant of a closed neural tube defect characterized by the longitudinal division of the spinal cord, resulting in the formation of duplicated neural tubes or two hemicords within a single dural sleeve. This condition typically arises from anomalies within the spinal canal and congenital spinal deformities during embryogenesis, and is frequently comorbid with congenital scoliosis (CS)[5]. Studies by McMaster[6] and Bradford [7] have respectively indicated a 16% and 17% incidence of diastematomyelia among individuals with CS. Shen [8] identified SCM as the most prevalent spinal intradural anomaly in the context of CS. The occurrence of CS in SCM patients is documented to range from 52% to 79%[9-12]. Select the treatment plan based on the benefits and risks of alternative options[13]. Longitudinal spinal traction has gained consensus for improving flexibility and reducing correction risk in severe scoliosis[14-17]. Some scholars have reported favorable correction outcomes with two-stage correction and internal fixation following heavy traction for severe scoliosis[18-25]. It's believed that skull-femoral traction can effectively prevent excessive correction angles during traction, thereby avoiding spinal cord and important neurovascular damage[26,27]. In our department, skull-femoral traction followed by correction and internal fixation has yielded good results, with 82 cases completed, including 10 cases of accompanying split cord malformation, all without irreversible nerve damage. Therefore, a retrospective analysis was conducted on cases of severe scoliosis with split cord type I malformation treated with skull-femoral traction followed by two-stage correction, aiming to explore the safety and clinical efficacy of this treatment approach.

Evaluation of Research Methodology

1. Methodological Strengths:

Comprehensive Approach: The study employs a comprehensive methodology that includes detailed pre- and post-treatment assessments of various parameters such as spinal angles, physiological measurements (e.g., VC, FVC, FEV1), and biochemical markers (e.g., albumin, transferrin). This approach provides a thorough evaluation of the treatment's effects on both structural and functional outcomes.

Longitudinal Assessment: The use of multiple follow-up points (after traction, after corrective surgery, and at the last follow-up) enhances the reliability of the findings by capturing changes over time and assessing the sustainability of treatment outcomes.

Absence of Complications: The study reports no neurological impairments or significant complications related to the traction or osteotomy procedures, indicating careful procedural management and patient monitoring.

2. Areas for Improvement:

Sample Size and Diversity: The study's sample size of ten patients limits the generalizability of findings. Including a larger and more diverse patient cohort could strengthen the study's external validity and allow for subgroup analyses based on different patient characteristics.

Control Group or Comparative Analysis: Introducing a control group or comparative analysis with alternative treatment methods would provide a clearer basis for evaluating the specific benefits of skull-femoral traction followed by osteotomy correction compared to standard treatments.

Data Standardization: Ensure consistent data collection protocols across all measurements to minimize variability and enhance the reliability of comparisons over time.

3. Data Analysis:

Statistical Rigor: The statistical analysis appears appropriate for the study's objectives, utilizing descriptive statistics and measures of central tendency to summarize continuous data. However, considering the complexity of the data (e.g., longitudinal measurements), employing more sophisticated statistical methods such as repeated measures ANOVA or mixed-effects models could provide deeper insights into treatment effects over time.

In conclusion, while the study demonstrates methodological rigor in its approach to evaluating skull-femoral traction followed by osteotomy correction for severe spinal scoliosis with split cord malformation, there are opportunities for enhancement, particularly in sample size, comparative analysis, and data standardization, to further strengthen the validity and generalizability of its findings.

**Response:** Thank you for your thorough evaluation of our research methodology. We appreciate the recognition of our methodological strengths, and we have taken your suggestions for improvement into consideration. Here is how we plan to address each point:

Sample Size and Diversity:We acknowledge the limitation in our sample size and its impact on the generalizability of our findings. In future studies, we will aim to include a larger and more diverse patient cohort to enhance the external validity of our research.

Control Group or Comparative Analysis:Your suggestion to introduce a control group or comparative analysis is well-received. While our current study did not include a control group, we will design future studies to include comparative analyses with alternative treatment methods to better evaluate the specific benefits of our treatment approach.

Data Standardization:We agree that consistent data collection protocols are essential for minimizing variability and ensuring the reliability of our comparisons. We will implement standardized procedures for data collection in future research to improve the rigor of our methodology.

Data Analysis:We appreciate your comments on statistical rigor. While our current analysis utilized descriptive statistics suitable for our data, we will consider employing more sophisticated statistical methods such as repeated measures ANOVA or mixed-effects models in future studies to provide a deeper understanding of treatment effects over time.

We concur with your conclusion that our study demonstrates methodological rigor and that there are opportunities for enhancement. We are committed to strengthening the validity and generalizability of our findings in future research by addressing the areas for improvement you have highlighted.

Results

Areas for Improvement:

1. Clarity and Logic of Data Presentation:

Issue: Some sections of data presentation may be too compact, making it challenging for readers to follow, especially in describing different patient scenarios.

Recommendation: Consider reorganizing the data, such as through clearer segmentation to distinguish between different cases and treatment effects. Ensure each case description is clear and structured to facilitate reader comprehension of the study's progression.

2. Statistical Analysis and Significance:

Issue: Descriptions regarding statistical significance should be more detailed and thorough to ensure readers have a clear understanding of the reliability of the results.

Recommendation: Clearly state the methods of statistical analysis and significance levels (e.g., P-values) in each results subsection, and discuss their implications for interpreting the study findings.

3. Use of Figures and Tables:

Issue: The results section could benefit from additional figures and tables to enhance the visual representation of data, aiding readers in quickly and intuitively grasping key findings.

Recommendation: Consider adding relevant figures and tables to the results section to showcase primary data and trends. This will highlight key results and improvement trends from the study.

**Response:** we sincerely appreciate the time and effort you have put into reviewing our manuscript. We have carefully considered your suggestions in the results section, particularly regarding the clarity of data presentation, the detail of statistical analysis, and the recommendations for using charts.

Clarity and logicality of data presentation:

We recognize your concern about the possibility of data presentation being too tight. However, we believe that the current organizational approach is aimed at maintaining the simplicity and directness of the results section, which helps readers quickly understand the main findings of our research. Our data presentation is based on careful consideration of the logic and coherence of the results, aimed at avoiding redundancy and excessive segmentation, which may distract readers' attention.

Statistical analysis and significance:

Thank you for your suggestions on statistical analysis and significance description. The statistical methods and reporting methods we have chosen are designed to match the scientific objectives and data characteristics of our research. We believe that the statistical information provided is sufficient to support our conclusions, and we have explicitly discussed the interpretation of these statistical results for the research findings in the text.

The use of charts and tables:

We appreciate your suggestion to add charts and tables to enhance the visualization of data. Although we agree that charts can provide intuitive data display, we choose to use them only when necessary to avoid overly complicating the results section. We believe that the charts and tables in the current manuscript are sufficient to convey key results and trends.

We believe that our manuscript provides clear, accurate, and meaningful research results in its current form, and has been presented in a reader friendly manner. We hope you can understand our position and acknowledge our presentation of the results section.

Discussion

Clarity of Logic and Structure:

Issue: The viewpoints and research findings in the discussion could be more clearly organized and presented, especially in describing different cases and treatment outcomes.

Recommendation: Consider reorganizing the discussion content to ensure there is a clear logical flow between each viewpoint and research result. Enhancing overall coherence with clearer paragraph structures and topic sentences would facilitate easier understanding and follow-up of the discussion's progression.

**Response:** Thank you for your insightful comments and suggestions regarding the organization of the Discussion section in our manuscript. We have given careful consideration to your recommendation to reorganize the content for improved clarity and logical flow. After a thorough review of our current manuscript structure, we believe that the existing organization effectively communicates our research findings and the implications of our study. The sequence of topics and the presentation of cases and outcomes are intentionally ordered to reflect the progression of our research and the narrative that supports our conclusions. We have provided detailed explanations and data in the Discussion to ensure that each point is well-supported and comprehensive. The current structure allows readers to follow the development of our argument and understand the context and significance of our findings. While we appreciate the opportunity to enhance the manuscript, we do not believe that additional reorganization is necessary at this stage. We trust that the manuscript's clarity and coherence are sufficient for the readership of the journal. We are, however, open to further considerations and will address any specific concerns that may arise during the peer review process. Thank you once again for your valuable feedback.

Sincerely,

Tao Guo
